# A survey of visualization tools for biological network analysis

**DOI:** 10.1186/1756-0381-1-12

**Published:** 2008-11-28

**Authors:** Georgios A Pavlopoulos, Anna-Lynn Wegener, Reinhard Schneider

**Affiliations:** 1 Structural and Computational Biology Unit, EMBL, Meyerhofstrasse 1, Heidelberg, Germany

## Abstract

The analysis and interpretation of relationships between biological molecules, networks and concepts is becoming a major bottleneck in systems biology. Very often the pure amount of data and their heterogeneity provides a challenge for the visualization of the data. There are a wide variety of graph representations available, which most often map the data on 2D graphs to visualize biological interactions. These methods are applicable to a wide range of problems, nevertheless many of them reach a limit in terms of user friendliness when thousands of nodes and connections have to be analyzed and visualized. In this study we are reviewing visualization tools that are currently available for visualization of biological networks mainly invented in the latest past years. We comment on the functionality, the limitations and the specific strengths of these tools, and how these tools could be further developed in the direction of data integration and information sharing.

## Introduction

Bioinformatics has evolved and expanded continuously over the past four decades and has grown into a very important bridging discipline in life science research. The quantities of data obtained by new high-throughput technologies, including micro or Chip-Chip arrays, and large-scale "OMICS"-approaches, such as genomics, proteomics and transcriptomics, are vast and biological data repositories are growing exponentially in size. Furthermore, every minute scientific knowledge increases by thousands pages and to read the new scientific material produced in 24 hours a researcher would take several years. To follow the scientific output produced regarding a single disease, such as breast cancer, a scientist would have to scan more than a hundred different journals and read a few dozens papers per day.

The underlying data sets show a growing complexity and dynamics and are produced by numerous heterogeneous application areas. The integration of heterogeneous types of data is therefore gaining in importance. Currently different biological types of data, such as sequences, protein structures and families, proteomics data, ontologies, gene expression and other experimental data are stored in distinct databases. Existing databases or data collection can be very specialized and often they store the information using specific data formats. Many of them also contain overlapping but not exactly matching information with other databases, which introduces another hurdle to combine the information. In order to gain insights into the complexity and dynamics of biological systems, the information stored in these data repositories needs to be linked and combined in efficient ways. To address these issues, data integration became a main issue in the past years [1].

The challenge lies very often in the analysis of a huge amount of data to extract meaningful information and use them to answer some of the fundamental biological questions. Given the heterogeneity and the sheer amount of data it is a challenge to detect the relevant information and to provide a way to communicate the findings of the researcher in an efficient and appropriate way.

The human brain has evolved remarkable visual processing capabilities to analyze patterns and images. Thus, an interactive visual representation of information together with data analysis techniques is often the method of choice to simplify the interpretation of data. A wide variety of tools was developed over the past years that map data on 2D graphs to visualize biological interactions or relationships between bio-entities.

Graphs, as specified by graph theory, represent biological interactions in the form of extensive networks consisting of vertices, denoting nodes of individual bio-entities, and edges, describing connections between vertices. In the simplest example two vertices are linked by only one relationship, but connections can express different types of relationships between two elements, such as an evolutionary relationship, the existence of a shared protein domain, the fact that they belong to the same protein family or that two genes that are co-expressed in an experiment. Biological systems are complex and interwoven and in most cases single-line connections are insufficient to capture the whole range of information contained in a network, because components are often linked by more than one type of relationship. Two proteins might be connected because they act as part of the same protein complex, show similarities in their functional annotation, co-occur under certain conditions or are known to be evolutionary related. In such cases visualization tools based on multi-edged networks offer the possibility to link two vertices by multiple edges, every edge having a different meaning and information value.

In this review we describe tools which try to simplify the analysis and interpretation of biological data by transforming the raw data into logically structured and visually tangible representations. The goal of all of them is to find patterns and structures that remain hidden in the raw unstructured datasets.

Since the wealth of existing visualization tools makes an exhaustive collection and in-depth discussion of all available software tools impossible, we present a selection of several network viewers, which are broadly applicable. Our survey covers tools invented mainly over the past five years and discusses some of their advantages and shortcomings in order to aid researchers in choosing the most suitable visualization tool for their studies. Finally, we will highlight crucial gaps in the landscape of data visualization, discuss how existing tools could be improved to fill these gaps and lay out the perspectives and goals for the next generation of visualization tools.

## A survey of network visualization tools

In the following section we discuss several widely used network visualization tools that have been developed over the past years. The presented tools are selected so as to cover the range of different functionalities and features crucial for data analysis and visualization. While the discussed tools are all broadly applicable, we will highlight their respective strengths and weaknesses if any, and comment on their specific features. Tools for analyzing protein-protein interactions, pathways, gene networks, heterogeneous networks or tools for studying evolutionary relationships between proteins have a very different scope and require a separate and much more detailed analysis which goes beyond the scope this review. Instead we try to provide a broad overview that can help guide users, especially those that are novices to the field of data visualization, towards the most appropriate tool for their research question and type of data.

Criteria for the assessment of visualization tools include power, efficiency and quality of network visualizations produced, the compatibility with other tools and data sources, the analytical functionalities offered (with specific focus on pattern recognition, data integration and comparison), limitations in terms of data quantity, broad applicability and user-friendliness. Following the detailed evaluation with respect to these criteria, a one sentence summary highlights the particular strengths of each tool.

### Medusa [2]

Medusa is a Java application and available as an applet. It is an open source product under the GPL license.

#### Visualization

Based on the Fruchterman-Reingold [3] algorithm, Medusa provides 2D representations of networks of medium size, up to a few hundred nodes and edges. It is less suited for the visualization of big datasets. Medusa uses non directed, multi-edge connections, which allows the simultaneous representation of more than one connection between two bioentities. Additional nodes can be fixed in order to facilitate pattern recognition and spring embedded layout algorithms help the relaxation of the network. Medusa supports weighted graphs and represents the significance and importance of a connection by varying line thickness.

#### Compatibility

Medusa has its own text file format that is not compatible with other visualization tools or integrated with other data sources. The input file format allows the user to annotate each node or connection.

#### Functionalities

Medusa is highly interactive. It allows the selection and analysis of subsets of nodes. A text search, which supports regular expressions, can be applied to find nodes. The status of a network can be saved and reloaded at any time but medusa is currently not connected to any data source.

#### Strength

The tool was developed mainly to show multi-edge connections where each line represents different concepts of information. Medusa is optimized for protein-protein interaction data as taken from STRING [4] or protein-chemical and chemical-chemical interactions as taken from STITCH [5].

### Cytoscape [6]

Cytoscape is a standalone Java application. It is an open source project under LGPL license.

#### Visualization

Cytoscape mainly provides 2D representations and is suitable for large-scale network analysis with hundredth thousands of nodes and edges. It can support directed, undirected and weighted graphs and comes with powerful visual styles that allow the user to change the properties of nodes or edges. The tool provides a variety of layout algorithms including cyclic and spring-embedded layouts. Furthermore, expression data can be mapped as node color, label, border thickness, or border color.

#### Compatibility

Cytoscape comes with various data parsers or filters that make it compatible with other tools. The file formats that are supported to save or load the graphs are SIF, GML, XGMML, BioPAX, PSI-MI, SBML, OBO. Cytoscape also allows the user to import mRNA expression profiles, gene functional annotations from the Gene Ontology (GO) and KEGG. Users can also directly import GO Terms and annotations from OBO and Gene Association files.

#### Functionalities

It is highly interactive and the user can zoom in or out and browse the network. The status of the network as well as the edge or node properties can be saved and reloaded. In addition, Cytoscape comes with a network manager to easily organize multiple networks. The user can have many different panels that hold the status of the network at different time points which makes it an efficient tool to compare networks between each other. It also comes with efficient network filtering capabilities. Users can select subsets of nodes and/or interactions and search for active subnetworks or pathway modules. It incorporates statistical analysis of the network and it makes it easy to cluster or detect highly interconnected regions.

#### Strength

Cytoscape main purpose is the visualization of molecular interaction networks and their integration with gene expression profiles and other data. It also allows the user to manipulate and compare multiple networks. Many plug-ins created by users are available and allow more specialized analysis of networks and molecular profiles.

### BioLayout Express3D [7]

BioLayout Express^3D ^is written in Java 1.5 and it uses the JOGL system for OpenGL rendering. It is released under the GNU Public License (GPL). A medium or higher range graphics card is necessary to run the software.

#### Visualization

BioLayout Express^3D ^is a tool for layout, visualization and clustering of large scale networks in both 3D and 2D. It supports both unweighted and weighted graphs together with edge annotation of pairwise relationships. It mainly employs the Fruchterman-Rheingold layout algorithm for 2D and 3D graph positioning and display of the network. A variety of colour schemes render the network more informative and clusters can be easier visualized. Since BioLayout Express^3D ^uses a graphics renderer it is limited in the size of networks it can process.

#### Compatibility

It comes with a very simple input file format requiring the user to only provide a list of connections. The tool is compatible with Cytoscape and it supports layout, expression, yEd GraphML and sif file formats. It is currently not connected with data sources but in the next versions SBML support will make it compatible with various currently available databases.

#### Functionalities

BioLayout Express^3D ^is highly interactive and the user can switch between 2D and 3D representations. Users can move around the current view, zoom in/out, rotate or move the network. In the latest version, the Markov Clustering algorithm (MCL) has become an integral part of BioLayout Express^3D ^for clustering analysis. In this way data are automatically separated in distinct groups labeled by different color schemes.

#### Strength

BioLayout *Express*^3D ^offers different analytical approaches to microarray data analysis.

### Osprey [8]

Osprey is a standalone application running under a wide range of platforms. It can be licensed for non commercial use and but source code is currently not available.

#### Visualization

Osprey provides 2D representations of directed, undirected and weighted networks. It is not efficient for large scale network analysis but it provides various layout options and ways to arrange nodes in various geometric distributions. The layouts range from the relax algorithm over a simple circular layout to a more advanced Dual Spoked Ring layout that displays up to 1500 – 2000 nodes in a easily manageable format. The user can change the size and the colours of most Osprey objects such as edges, nodes, labels, and arrow heads.

#### Compatibility

Data can be loaded into the tool either using different text formats or by connecting directly to several databases, such as the BioGRID [9] or GRID (General Repository of Interaction Datasets) database. In addition to its own Osprey file format the tool can also load Custom Gene Network and Gene List formats, making Osprey compatible with other tool relying on the same file formats. Osprey networks can be saved in SVG, PNG and JPG format.

#### Functionalities

The tool provides several features for functional assessment and comparative analysis of different networks together with network and connectivity filters and dataset superimposing. Osprey also has the ability to cluster genes by GO Processes. Network filters can extract biological information that is supplied to Osprey either by the user or by instructions inside the GRID dataset. Connectivity filters identify nodes based on their connectivity levels. These are Minimum, Iterative Minimum and Depth. Finally, Osprey includes basic functions such as selecting and moving individual nodes or groups of nodes or removing nodes and edges.

#### Strength

With its various filtering capabilities, Osprey is a powerful tool for network manipulation. The ability to incorporate new interactions into an already existing network might be considered the tool's biggest asset.

### ProViz [10]

ProViz is a standalone open source application under the GPL license.

#### Visualization

It comes with both 2D and pseudo-3D display support to render data. It can manipulate single graphs in large-scale datasets with millions of nodes or connections. Leveraging the Tulip [11] drawing package, it generates appealing 3D visualizations. ProViz predominantly relies on the GEM [12] force based graph layout algorithm which facilitates the identification of key points in a network of interactions. In addition the tool also offers a circular and a hierarchical layout, which improve the detection of metabolic pathways or gene regulation networks in large datasets. ProViz is ideal to gain a first overview of networks because it allows fast navigation through graphs.

#### Compatibility

Graphs are saved and loaded in Tulip, PSI-MI [13] and IntAct [14] formats. Networks can also be exported in PNG format. ProViz addresses queries directly to the IntAct database even though it comes with support of some standard file formats, established in systems biology, like PSI-MI (see below).

#### Functionalities

Subgraphs that are produced by selection, filtering or clustering methods and can be automatically organized into views. With ProViz it is possible to annotate each node and each edge with comments or merge different datasets into a single graph. Users can also enrich the networks by querying available online databases. ProViz uses a controlled vocabulary on bio-molecules and interactions, described in XML format.

#### Strength

ProViz has its strength in the area of protein – protein interaction networks and their analysis using arbitrary properties, like for example annotations or taxonomic identifier. Its plug-in architecture allows a diversification of function according to the user's needs.

### Ondex [15-17]

Ondex is a standalone freely available open source application.

#### Visualization

Ondex provides 2D representations of directed, undirected and weighted networks. It can handle large scale networks of hundred thousands of nodes and edges. It also supports bidirectional connections, which are represented as curves. Moreover, different types of data are separated by placing them in different disks-circles interconnected between each other.

#### Compatibility

Data may be imported through a number of 'parsers' for public-domain and other databases, such as TRANSFAC [18-20], TRANSPATH [21,22], CHEBI [23], Gene Ontology [24], KEGG [25-27], Drastic [28], Enzyme Nomenclature-ExPASy [29], Pathway Tools [30], Pathway Genomes (PGDBs), Plant Ontology[31], and Medical Subject Headings Vocabulary – MeSH [32]. Graph objects can be exported to Cell Illustrator and XML formats. To reload or feed into other applications graph objects may be saved as ONDEX XML or an XGMML form.

#### Functionalities

Ondex integrates various filters that selectively add or remove connected nodes from the display according to user selectable rules of connectivity type like distance, level or equivalence. A SubTreeFilter can extract a tree-like sub-graph from a given node. Furthermore, the tool comes with a KnockOutFilter which can be used to determine the most important nodes at any given level. Data for integration is modeled as a suitable framework of concepts (such as gene, pathway, and protein) and relations (such as 'belongs_to', 'is_a') describe the mapping between them. In addition, a powerful filter is available to import microarray expression level data to globally analyze the relations between the different genes being expressed.

#### Strength

Ondex main strength is the ability to combine heterogeneous data types into one network. It is suitable for text mining, sequence and data integration analysis.

### PATIKA [33]

PATIKA (**P**athway **A**nalysis **T**ools for **I**ntegration and **K**nowledge **A**cquisition) is a web based non-open source application publicly available for non-commercial use. It has its own license.

#### Visualization

It provides 2D representations of single or directed graphs. There are no limitations regarding the size of the graphs. It offers a very intuitive and widely accepted representation for cellular processes using directed graphs where nodes correspond to molecules and edges correspond to interactions between them. Even though the implemented variety of layout algorithms is rather limited, PATIKA is able to support bipartite graph of states and transitions. It represents different types of edges: product edges, where the source and target nodes of a product edge define the transition and a product of this transition, activator edges, where the source and target nodes of an activator edge define the activating state and the transition that is activated by this state, inhibitor edges where the source and target nodes of an activator edge define the inhibiting state and the transition that is inhibited by this state and substrate edges where the target and source nodes of a substrate edge define the transition and a substrate of this transition, respectively.

#### Compatibility

PATIKA integrates data from several sources, including Entrez Gene [34], UniProt [35], PubChem [36], GO [24], IntAct [14], HPRD [37], and Reactome [38,39]. Users can query and access data using PATIKA's webquery interface, and save their results in XML format or export them as common picture formats. BioPAX and SBML exporters can be used as part of Patikas Web service.

#### Functionalities

The user can connect to the server and query the database to construct the desired pathway. Pathways are created on the fly, and drawn automatically. The user can manipulate a pathway through operations such as add new state or remove an existing transition, edit its contents such as the description of a state or transition or change the graphical view of a pathway component.

#### Strength

PATIKA is a tool for data integration and pathway analysis. It is an integrated software environment designed to provide researchers a complete solution for modeling and analyzing cellular processes. It is one of the few tools that allows to visualize transitions efficiently.

### PIVOT [40]

PIVOT is a Java application, free for academics. It comes with its own license agreement.

#### Visualization

It projects everything in 2D and it uses single non directed lines to show relationships between bioentities. It is not limited to the size of data it can present. Overall the variety of incorporated layout algorithms is limited, but PIVOT employs specific layout algorithms for visualizing families.

#### Compatibility

Configured to work with proteins from four different species (human, yeast, drosophila and mouse), present functional annotations, identification of homologs from the four species, and links to external web information pages. The protein data are stored in an MS-Access file, easily modifiable by the users to enter their own data.

#### Functionalities

PIVOT can expand the network to display all proteins up to a specified distance, detect the shortest path of interactions or unfold the relationships among "distant" proteins, which respond similarly under a experiment's conditions. It can highlight dense areas of the map and use a window to visualize a subarea of a big networks. It is rich in features that help the users navigate and interpret the interactions map, as well as graph-theory algorithms for easily connecting remote proteins to the displayed map.

#### Strength

PIVOT is best suited for visualizing protein-protein interactions and identifying relationships between them, for example homologs.

### Pajek [41]

Pajek is a standalone application. It is not an open source application but it is free for non-commercial use. It runs under Windows OS only.

#### Visualization

It offers 2D representations and pseudo3d representations and supports single, directed and weighted graphs. Pajek is suitable for large scale networks with thousands or even million of nodes and vertices. It comes with a great variety of layout options like circular layout using partitions, circular layout using permutation or circular layout using random coordinates layout algorithms. Direct forcing and energy free layout algorithms [42], such as the Kimura-Kawai [43] and Fruchterman-Reingold [3] with free or fixed points are also included. It can also separate data into layers, which allows the display of hierarchical relationships. Pajek's ability to visualize multi-relational networks, networks between two disjoint sets of vertices and temporal networks make it one of the few tools that can handle dynamic graphs and reveal how networks change over time.

#### Compatibility

It comes with its own input file format, which is not compatible with commonly used XML formats. Neither is Pajek connected with any biological data sources. The status of the network can be saved any time and it allows export of information in EPS, SVG, X3D and VRML formats.

#### Functionalities

The tool is highly interactive and it incorporates many clustering methods, of which many are not very widely used however. It supports abstraction by (recursive) decomposition of a large network into several smaller networks and it implements a selection of efficient subquadratic algorithms for analysis of large networks. Pajek can detect clusters (components, neighborhoods of 'important' vertices, cores, etc.) in a network, extract vertices that belong to the same clusters and show them separately, shrink vertices in clusters or show relations among clusters.

#### Strength

Pajek's main strength is the variety of layout algorithms which greatly facilitate exploration and pattern identification within networks.

### Summary

The field of data visualization currently faces three major challenges: ever increasing quantity of data to be visualized and analyzed, integration of heterogeneous data and the representation of multiple connections between nodes with heterogeneous biological meanings.

As the survey shows, each visualization tool has specific features and thus the tools vary in how they address the outlined challenges. When heterogeneity of data is the major challenge, integrative tools like Ondex, Pivot or Medusa offer possible solutions. When the sheer mass, but less the heterogeneity, poses a problem, tools with high resolution and good scaling functionalities, like Cytoscape or BioLayoutExpress^3D ^are well equipped to help overcome these limitations. When working with systems biology data, that is highly interconnected and often linked by multiple biological relationships, Medusa and other tools featuring multi-edged networks are the most suitable. Pajek on the other hand is ideal for pattern recognition and to study the properties, such as density, centrality and frequency of nodes, of simple networks with single connections. For biological questions with a comparative focus Osprey is advisable.

Partly, more specialized tools are VisLink [44], KnowledgeEditor [45], Java editor for biological pathways [46], Pathway Studio [47], GenePath [48], PathScout [49], MAPMAN [50], MetaShark [51], CnPlot [52], CLANS [53], GSCope [54], BicOverlapper [55], Celestial3D [56], MetaNetter[57], HyperGraph, VANTED [58], Arena3D, SpectralNET [59], TouchGraph Google Browser, VisLink [44], SpectralNET [59], CnPlot [52]. Before this new generation of visualization tools became available Otter [60], Plankton [61], GraphViz, Tulip[11], Negopy[62], KrackPlot [63], and MultiNet [64], have been the tools of choice.

## Standard network file formats

One of the issues that most of the visualization tools have to address first is the complexity of the input data. Currently most of the network graph viewers come with their own input file formats to load and store the networks. This makes it difficult to use various tools for the same dataset since the user has to reformat the dataset every time according to the specific tool. This can be a strong limitation in cases where one would like to take advantage of the complementary strengths of different tools. We found that the missing interoperability between tools is one crucial bottleneck in exploring the variety of available methods.

Moving towards data integration, a number of common file formats and standard languages to store biological information have been introduced. Datasets that are stored in a standardized format can easily be incorporated into a visualization tool that supports the same format without requiring reprogramming nor understanding of the file format itself. Computer science has identified Extensible Markup Language (XML) as an appropriate format for data visualization because it is readable by both, computers and humans. XML stores information in the form of hierarchical tree structures, which allows fast and efficient searching by humans as well as machines. One other main factor why XML is in many cases a very suitable format is the platform-independent text-based format, which supports Unicode and is based on international standards. A further advantage of XML is the forward and backward compatibility which are easy to maintain. Of course one of the drawbacks of XML schemas is that the inherent redundancy may affect application efficiency due to higher storage, transmission and processing costs.

In the following section we discuss the most widely used file formats and standard languages in bioinformatics and chemoinformatics. Many open-source XML-based languages, most notably BioPAX, SBML and PSI-MI, rely on a leveled approaches, meaning that they contain various levels of complexity and specificity. It is a choice of the user to specify the level required to represent the information in their dataset.

### BioPAX [65]

This "pathway language" is a collaborative effort to create a computer readable data exchange format for biological data. The language was developed to allow distribution, sharing and exchange of information between pathway databases in a standard format by using a specific controlled vocabulary for tagging. BioPAX is based on an ontology of concepts with attributes, which allows to make a more explicit use of the relations between concepts compared to other standards. It is most suitable for the description of protein-protein interactions, genetic interactions, gene regulatory, metabolic and signaling pathways. BioPAX is being developed in a series of levels incorporating different features in each round. The current version has the focus on metabolic networks and molecular interaction networks, were the next development level is trying to implement gene and DNA interactions, signal transduction and genetic interactions. BioPAX is the most expressive language and is based on a rich hierarchy, which as a trade-off can result in a high degree of computational complexity. Being a comparatively new language BioPAX is not yet supported by the majority of tools presented here.

### SBML [66,67]

The acronym SBML stands for Systems Biology Markup Language and is a machine-readable format for describing qualitative and quantitative models of biochemical networks. The current version of SBML focuses on models for the analysis and simulation of basic biochemical networks. The next release will additionally incorporate the concept of model composition, the description of molecule complexes, layout information and spatial characteristics of the models. Many libraries and tools are available for parsing and editing SBML texts. Furthermore, several converters exist to convert SMBL into BioPAX and vice versa. Having started of as a language to describe biochemical reactions, SBML is now widely accepted and supported by over 100 different software systems worldwide, including systems for modeling and simulation, drawing and visualization tools and databases such as KEGG and BioCyc [68].

### PSI-MI [13]

PSI-MI stands for Proteomics Standards Initiative Interaction and is a machine readable format intended for the exchange, comparison and verification of proteomics data. There are many tools available for viewing and converting PSI-MI data. The main focus is the definition of molecular interactions such as protein-protein interactions, rather than the description of complete cellular models.

### CML

CML [69,70] is the acronym for Chemical Markup Language and is a language mainly developed to describe chemical concepts and information about molecules, reactions, spectra and analytical data, computational chemistry, chemical crystallography and materials.

### CellML

Cell Markup Language [71] is an XML-like machine-readable language mainly developed for the exchange of computer-based mathematical models. CellML was originally developed for biological applications, but later proved to be applicable also to other disciplines. It can incorporate mathematical metadata by leveraging existing languages, including MathML [72] and RDF [73,74]. CellML can hold information about data models, mathematic formulas and equations as well as metadata.

### RDF

The Resource Description Framework, RDF [73,74], is a language for the representation of information about resources on the World Wide Web. Since the World Wide Web moves towards semantic web structures, RDF was designed as a machine-readable XML-like language that describes networks. RDF tags employ a controlled RDF vocabulary. The idea behind RDF is the identification of Uniform Resource Identifiers (URIs) and the description of resources in terms of simple properties and property values.

BioPAX, SMBL and PSI MI are the three languages most applicable to biological data [75]. Out of them BioPAX has the richest hierarchy due to the advanced tagging vocabulary and has the most general approach. It spans a broad range of biological data including genetic interactions, interaction networks, small molecules as well as regulatory and metabolic pathways. PSI-MI is ideal to handle experimental data like molecular interactions and interaction networks. SBML, on the other hand, is better suited for the description of relationships and is mostly used in simulations. It is the language of choice when it comes to rate formulas and biochemical reactions. A more detailed, comparative evaluation of these most common three file formats can be found in a recent study [76].

## Discussion

Nowadays, biological projects and experiments become more complex and bigger in scope and data that are produced are magnitudes higher than in the past. The increasing use of high-throughput technologies multiplies the amount of data generated per experiments and rapidly increases the sizes of the databases. In the analysis step we can identify the visualization of data as an already major bottleneck. The pure amount of data and their heterogeneity pose a challenge for efficient visualization tools. The main goal of the visualization tools should be the intuitive representation of data to provide an efficient interpretation and to allow a hypothesis driven planning of the next experiment.

The tools represented in this review are applicable to a wide range of problems and their distinct features make them suitable for a wide range of applications. However, despite the continuous improvement of visualization algorithms, most existing visualization methods reach limitations in terms of user friendliness when thousands of nodes have to be analyzed and visualized. The majority of tools discussed in this review can cope with datasets of up to about 5000 nodes without compromising too severely on speed and user friendliness. Yet, results of large-scale systems biology experiments frequently exceed hundred thousands of data points today, and many existing visualization tools are not able to cope with these demands calling for a new generation of visualization tools.

In order to improve the scaling for big datasets most layout algorithms follow a heuristic approach instead of an exhaustive implementation. Despite the wealth of existing algorithms, the layout problem still remains one of the crucial bottlenecks in network visualization. Faster and more efficient algorithms are needed to bring especially large-scale networks into a form that can be easily understood and interpreted by the human brain. One way to circumvent the problem is parallelization of layout, clustering and graph theory algorithms that they can handle large networks. One solution could be the implementation of web services or libraries that outsource most of the computational effort and calculations to distant, powerful machines that can run many parallel jobs would greatly speed up the process of visualization and reduce the computational load on local computers. Another solution would be the way that these layout algorithms are written to be written in such a way that they can take advantage of the multi-core CPU or GPU technologies.

In addition, the extension of layout algorithms to encompass a third dimension would be one central step towards a new generation of visualization tools. This becomes more important in cases like pathway or heterogeneous data sets visualization. The extra dimension would allow a clearer structure and less cluttered views and could strongly facilitate a better navigation within the network. The extension of layout algorithms into three dimensions could thus render the representation of large-scale networks much more efficient, because 3D space minimizes the chance of crossover between two edges.

The third dimension would also offer an opportunity to fill a crucial gap in network data visualization; that is the representation of time. Currently, most network tools do not attempt to visualize time series data [42] and thus only produce a static snapshot of all the interactions happening in dynamic systems. Introducing the parameter time as an extra dimension into network visualization tools would thus achieve a more complete picture of complex and highly dynamic biological systems. Being able to investigate the dynamics of a system could provide breakthroughs in fields such as pathway analysis or the observation of interaction at different cell cycle time points.

The rapid growth of data calls for the incorporation of powerful filters into visualization tools. Filters that reduce the noise in a dataset and restrict the user's attention to a core set of nodes of a particular interest could greatly improve visualization. Similarly, more efficient and interactive graphical user interfaces (GUIs) would allow the user to visualize and explore relevant sub networks or limited areas of a whole network without having to sieve through vast data masses. To increase the performance of visualization tools further, efficient handling and allocation of memory is essential. This can be achieved by loading only the necessary parts of the graph into memory. In this way, the amount of data and the taxonomies that can be visualized can be rapidly increased. Of course, Graphical Process Units (GPUs) hardware performance increases over time, something that allows visualization tools to employ more resource demanding algorithms like those handling advanced graphics calculations.

The future generation of visualization tools should aim to reduce the gap between analysis and visualization. Most existing visualization tools only incorporate a limited number of data analysis functionalities, making it necessary to constantly switch between different applications. The user has to be aware of the variety of tools that are suitable to analyze his data and must switch between them. Information and data sharing between different tools has become a much simpler task due to standard file formats, which should be supported by newly developed visualization tools. Standard formats that are applicable to many different data types will be key features for the growing need to integrate heterogeneous data into a network. A true marriage of analysis and visualization, however, cannot be achieved merely by the support of multiple, standard file formats. Instead, future visualization applications should directly include several of the analytical functionalities that are available in the presented tools.

Ideally, the next generation of visualization tools should be able to present very heterogeneous data coming from databases, experiments and text-mining applications. They need to be able to visualize multi-edged networks, incorporate widely used clustering techniques, pattern recognition algorithms and statistical analysis methods. While technology evolves the visualization tools could explore the wider use of autostereoscopic 3D displays, which allow seeing three-dimensional images without the need of special glasses. A visualization tool designed to integrate most of the aforementioned functionalities would greatly simplify large-scale research in molecular biology and would significantly cut down time and effort spent on data processing and analysis.

In summary and to provide some concrete solutions to visualization tool challenges we suggest the following:

• Visualization should be able to load and save data using worldwide standard file formats.

• Incorporation of appropriate statistical analysis of the networks.

• Algorithms that allow comparative analysis of different networks.

• Implementation of libraries and services that allow layout algorithms to run in distant powerful computers.

• Efficient layout algorithms that are able to use multi-core CPU technology.

• Algorithms that implement rendering and graphical calculations in GPU.

• Expansion of layout algorithms into 3D space especially for the visualization of pathway or heterogeneous data.

• Visualization of the network behavior and its changes over time. Such animations are currently possible using Flash technologies.

## Competing interests

The authors declare that they have no competing interests.

## Authors' contributions

GAP was the main author of the paper. He collected all of the necessary information for the review article and he combined the knowledge by comparing different tools between each other to find their their strengths and their weaknesses.  A-LW was the main writer of the manuscript.  RS was the scientific supervisor of this study. He contributed strongly in correcting and writing parts of the manuscript.
